# The Story of the Insane from Year to Year

**Published:** 1902-07-12

**Authors:** 


### THE STORY OF THE INSANE FROM YEAR TO YEAR.

Fourteenth Annual Series.

### HANTS COUNTY ASYLUM AT FAREHAM.

On January 1, 1900, this asylum contained 1,077 patients, and at the end of the year the number had risen to 1,092, There were 199 first admissions, 28 readmissions, 73 discharged recovered, 9 discharged relieved, and 104 deaths. The average number resident during the year was 1,087. Calculated on the admissions, the recoveries stand at 32·1 per cent., and the deaths at 9·5 per cent. on the average number resident. Of the deaths during the year, 37 were caused by cerebral and spinal diseases, 8 by consumption, and 2 by pneumonia. Of the year's admissions, 9 owed their insanity to adverse circumstances, 5 to religion, 41 to intemperance in drink, 48 to hereditary tendency, 38 to previous attacks, and 25 to congenital defect. These numbers show that drink and heredity account for 89 of the 227 admissions, and high as this is there can hardly be any doubt that it would be considerably increased if sufficiently close investigations could be made. The asylum was full on the date of the report, and the additions were not ready for occupation, and Dr. Worthington said he "would be very sorry to hazard an opinion as to the date when they will eventually be fit for use." When finished there will be room for 1,166 beds. The committee "tender their thanks to Dr. Worthington, the other medical officers, and the staff in general, for the excellent way in which they have performed their various duties." The rate of maintenance was 8s. 9d. per head per week.

### WEST SUSSEX ASYLUM AT CHICHESTER.

An increase from 479 to 516 patients has to be chronicled for the year 1900. There were 86 first admissions, 23 readmissions, 26 recoveries, and 39 deaths. The average number resident was 505. Excluding transfers, the recoveries stand at 24·76 per cent. of the admissions, and the deaths 7·72 per cent. of the average number resident. Of the deaths, 19 were caused by cerebral and spinal diseases, 2 by consumption, and 6 by other lung diseases. Various moral causes are supposed to account for the insanity in 18 of the year's admissions, and among the physical causes we note the usual preponderance of hereditary influence and intemperance in drink, these two being responsible for 57 cases, that being about 50 per cent. of the total number of admissions of the year. This blot on our civilisation is not confined to West Sussex. On the contrary, it is demonstrated in every asylum in England, and its very prevalence seems to be the reason of its escaping comment. Remarking on the number of old feeble cases sent to the asylum Dr. Kidd thinks that the growing tendency to have these patients certified is one cause of the alleged increase of insanity. It is an evil pretty freely discussed in some asylum reports, but not much attempt beyond discussion is made to lessen the evil. Another point which crops up rather often is the restlessness of the staff; and this is, considering the duties of attendants and nurses, not altogether to be wondered at; but it is enormously increased by the ease with which the attendant or nurse can find employment in some other asylum. Dr. Kidd says his "male staff is in a satisfactory condition." Let him be thankful for so much even although the female staff "display crude notions of discipline, a wilful disregard of obvious precautions, and a tendency to resign after correction with some vague idea of retaliation." The visiting committee "are glad to be able to record their sense of the services rendered to the County by the able discharge by Dr. Kidd of his various duties." A pension scheme has been under discussion of the committee, but has been postponed until something is done by Parliament. This is putting off one of the most important things in connection with asylums management until the Greek Kalends. The visiting committee may feel absolutely certain that the adoption of a satisfactory pension scheme would have a very decided effect in settling the best and thinking part of the staff. Besides, it is evident that the greater the number of asylums which voluntary pass a pension scheme the greater will be the likelihood of the passing of a pensions clause in any Bill which may ultimately be debated. The maintenance rate is very high, namely, 12s. 3d. per head per week.

